# Leptomeningeal Metastasis: The Role of Cerebrospinal Fluid Diagnostics

**DOI:** 10.3389/fneur.2019.00839

**Published:** 2019-08-20

**Authors:** Lena Bönig, Nora Möhn, Jonas Ahlbrecht, Ulrich Wurster, Peter Raab, Wolfram Puppe, Kurt-Wolfram Sühs, Martin Stangel, Thomas Skripuletz, Philipp Schwenkenbecher

**Affiliations:** ^1^Department of Neurology, Clinical Neuroimmunology and Neurochemistry, Hannover Medical School, Hanover, Germany; ^2^Department of Diagnostic and Interventional Neuroradiology, Hannover Medical School, Hanover, Germany; ^3^Department of Virology, Hannover Medical School, Hanover, Germany

**Keywords:** leptomeningeal metastasis, cerebrospinal fluid, cytological examination, malignancy, MRI, oligoclonal bands

## Abstract

**Background:** Metastatic spread into the cerebrospinal fluid (CSF) represents a severe complication of malignant disease with poor prognosis. Although early diagnosis is crucial, broad spectrums of clinical manifestations, and pitfalls of magnetic resonance imaging (MRI) and CSF diagnostics can be challenging. Data are limited how CSF parameters and MRI findings relate to each other in patients with leptomeningeal metastasis.

**Methods:** Patients with malignant cells in CSF cytology examination diagnosed between 1998 and 2016 at the Department of Neurology in the Hannover Medical School were included in this study. Clinical records, MRI findings and CSF parameters were retrospectively analyzed.

**Results:** One hundred thirteen patients with leptomeningeal metastasis were identified. Seventy-six patients (67%) suffered from a solid malignancy while a hematological malignancy was found in 37 patients (33%). Cerebral signs and symptoms were most frequently found (78% in solid vs. 49% in hematological malignancies) followed by cranial nerve impairment (26% in solid vs. 46% in hematological malignancies) and spinal symptoms (26% in solid vs. 27% in hematological malignancies). In patients with malignant cells in CSF MRI detected signs of leptomeningeal metastasis in 62% of patients with solid and in only 33% of patients with hematological malignancies. Investigations of standard CSF parameters revealed a normal CSF cell count in 21% of patients with solid malignancies and in 8% of patients with hematological malignancies. Blood-CSF-barrier dysfunction was found in most patients (80% in solid vs. 92% in hematological malignancies). Elevated CSF lactate levels occurred in 68% of patients in solid and in 48% of patients with hematological malignancies. A high number of patients (30% in solid vs. 26% in hematological malignancies) exhibited oligoclonal bands in CSF. Significant correlations between the presence of leptomeningeal enhancement demonstrated by MRI and CSF parameters (cell count, lactate levels, and CSF/Serum albumin quotient) were not found in both malignancy groups.

**Conclusion:** CSF examination is helpful to detect leptomeningeal metastasis since the diagnosis can be challenging especially when MRI is negative. CSF cytological investigation is mandatory whenever leptomeningeal metastasis is suspected, even when CSF cell count is normal.

## Introduction

Leptomeningeal metastasis is caused by malignant cells which infiltrate the cerebrospinal fluid (CSF) by hematogenous spread, endo-, or perineural dissemination along peripheral nerves, or by direct expansion of parenchymal cerebral metastases (1, 2). This devastating complication is diagnosed in 1–15% of patients with systemic malignancy but autopsy studies suggest a higher incidence as leptomeningeal metastasis was found in up to 20% of cancer patients suffering from neurological symptoms (1, 3). The incidence of leptomeningeal metastasis increased in the last decades due to improved systemic malignancy treatment, providing a larger time frame for this late stage complication to occur (4, 5). Nevertheless, spread of malignant cells into the CSF implies a limited prognosis with a median survival time of 2–6 months (1, 2, 5–7). Early diagnosis is needed to maintain quality of life and to improve survival time by treatments including intrathecal chemotherapy, systemic chemotherapy, and radiotherapy (8–10).

The clinical manifestation of leptomeningeal metastasis varies and comprises symptoms of brain, cranial nerve, and spinal cord involvement as CSF flow disseminates malignant cells throughout the entire central nervous system space (CNS) (8, 11). The diagnosis of leptomeningeal metastasis can be established by either evidence of malignant cells in CSF cytology or leptomeningeal enhancement demonstrated by magnetic resonance imaging (MRI) (12). Both methods are considered to be complementary in the diagnostic work-up as CSF cytology may be pathologic in some cases with normal imaging and vice-versa (12, 13).

Analysis of CSF cells is essential to exclude alternative diagnoses such as infectious or autoimmune diseases which can cause similar neurological symptoms and MRI findings (13, 14). Furthermore, CSF parameters such as total protein and lactate levels have been demonstrated to be prognostic factors for the disease course of leptomeningeal metastasis (7, 8, 12). In this study, we analyzed relationships between CSF findings, clinical manifestation, and MRI findings of patients with leptomeningeal metastasis with regard to the underlying malignancy.

## Methods

### Patients

**Figure 1:**
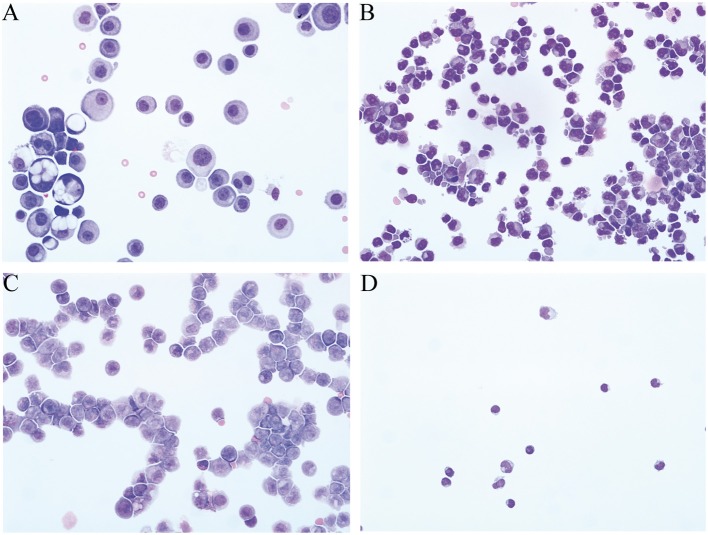
Representative CSF cytological findings of patients with leptomeningeal metastasis. **(A)** Large irregular shaped basophilic carcinoma cells with multiple nuclei and nucleoli in a patient with breast cancer. **(B)** Lymphoma cells with irregular size, pointed borders of the cytoplasm, and deep notches in the nuclei in a patient with NHL. **(C)** CSF cytology of a patient with acute myeloid leukemia with promyelocytes in different stages and myeloblasts. **(D)** Normal CSF cell profile.

Medical records of patients diagnosed with leptomeningeal metastasis who were admitted to the Department of Neurology of the Hannover Medical School between 1998 and 2016 were retrospectively identified. Patients were included in this study when malignant cells were found in CSF cytology examination (Figure 1). Clinical and laboratory data as well as MRI examination of brain and spinal cord were obtained. Analytical procedures are described in details in the section Appendix. Patients were categorized into two groups: patients with solid malignancies and patients with hematological malignancies. Findings of systemic tumor screening were considered when CSF cytological examination was confirmative for either malignant cells of hematological or solid tumor disease. The group of solid malignancies comprised lung cancer, breast cancer, gastrointestinal cancer, and other solid malignancies [melanoma, genitourinary cancer, solid brain tumor, and cancer of unknown origin (CUP)]. The group of hematological malignancies consisted of patients with lymphoid malignancies (systemic lymphoma, primary cerebral lymphoma, and multiple myeloma) and patients with myeloid malignancies (acute myeloid leukemia). The institutional ethic committee of the Hannover Medical School approved this investigation.

### Statistical Analysis

GraphPad Prism version 5.02 was used for statistical analysis. Continuous variables are given as medians and ranges. The D'Agostino–Pearson normality test was used to prove whether values were normally distributed. For normally distributed data one-way of variance and Bonferroni correction was used. In the absence of normal distribution Kruskal-Wallis and Dunns test was performed. Fisher's exact test was used to analyze categorical data. The level of statistical significance was set to 5%.

## Results

### Malignancy Characteristics

This study comprised a total of 113 patients with leptomeningeal metastasis. Details of malignancy characteristics are depicted in Table 1. In 76 patients (67%) a solid tumor was the cause for spread of malignant cells into the CSF. A hematological malignancy was found in 37 patients (33%) as the origin of malignant cells in the CSF.

**Table 1:** Clinical characteristics of patients with leptomeningeal metastasis.

**Characteristics**	**Age, years (range)**	**Females**	**Duration of neurological symptoms, days (range)**	**Primary tumor diagnosed after leptomeningeal metastasis**	**Time interval between diagnosis of primary tumor and leptomeningeal metastasis, months (range)**
Solid malignancies (*n* = 76)	59 (23–78)	64%	8 (1–180)	8%	15 (0–156)
Breast cancer (*n* = 26)	56 (37–73)	100%	18 (1–180)	0%	60 (1–156)
Lung cancer (*n* = 25)	62 (35–78)	52%	7 (1–00)	8%	9 (0–96)
Gastrointestinal cancer (*n* = 12)	63 (43–72)	42%	12 (1–60)	17%	14 (0–48)
Other malignancies (*n* = 13)	59 (23–76)	38%	7 (1–60)	15%	13 (0–96)
Hematological malignancies (*n* = 37)	58 (28–85)	41%	9 (1–145)	27%	12 (0–121)
Lymphoid malignancies (*n* = 31)	59 (35–85)	35%	9 (1–145)	29%	11(0–121)
Myeloid malignancies (*n* = 6)	48 (28–75)	66%	11 (1–21)	17%	17 (0–25)
*p* value	0.75	0.03	0.70	0.03	0.77

*Age, duration of neurological symptoms before diagnosis and time interval between diagnosis of primary tumor and leptomeningeal metastasis are presented by median and range*.

In the group of patients with solid malignancies, the most frequent tumor was breast cancer in 26/76 patients, followed by lung cancer in 25/76 patients, and gastrointestinal cancer in 12/76 patients. Other solid malignancies comprised four patients with melanoma, two patients with ovarian cancer, and one patient each with urothelial cancer, penis cancer, gliosarcoma, chordoma, astrocytoma, medulloblastoma, and one patient with CUP. The group of hematological malignancies consisted of lymphoid and myeloid malignancies. Of the 31 patients with lymphoid malignancies 8 patients had a primary CNS lymphoma and two patients were diagnosed with multiple myeloma. All 6 patients with a myeloid malignancy suffered from acute myeloid leukemia.

### Patients Demographics

The median age at diagnosis of leptomeningeal metastasis was 59 (23–78) years in patients with solid malignancies and 58 (28–85) years in patients with hematological malignancies (Table 1). More women were affected by a solid malignancy (64%) while in the group of hematological malignancies the male sex was prevailing (59%).

### Clinical Manifestations

In patients with a solid malignancy, the dominant neurological symptoms, and deficits that caused admission to hospital were cerebral involvement in 59 patients (78%), followed by cranial nerve impairment in 20 patients (26%), and spinal cord syndromes in 20 patients (26%) (Table 2). Symptoms of cerebral involvement consisted predominantly of headache, followed by disturbance of consciousness, brainstem/cerebellar signs, seizures, and vomiting/nausea.

**Table 2:** Presenting signs and symptoms of patients with leptomeningeal metastasis attributed to cerebral, cranial nerve, and spinal affection.

**Clinical manifestation**	**Solid malignancies (*n* = 76)**	**Hematological malignancies (*n* = 37)**	***p*-value**
Cerebral symptoms	78%	49%	0.003
Headache	34%	22%	0.20
Consciousness disturbance	24%	19%	0.64
Brainstem*/*Cerebellar signs	22%	8%	0.07
Seizures	18%	8%	0.17
Nausea/Vomiting	17%	5%	0.14
Cranial nerve symptoms	26%	46%	0.05
Nervus opticus (II)	35%	18%	0.29
Nervus oclumotorius (III)	20%	18%	1.0
Nervus trigeminus (V)	5%	24%	0.16
Nervus abducencs (VI)	20%	35%	0.46
Nervus facialis (VII)	20%	53%	0.05
Nervus vestibulocochlearis (VIII)	35%	12%	0.14
Nervus vagus (X)	10%	0%	1.0
Other cranial nerves	10%	6%	1.0
Spinal symptoms	26%	27%	1.0

Patients with hematological malignancies were almost twice as often affected by cranial nerve impairment than patients with a solid malignancy. However, cerebral symptoms and signs (49%) were prevailing with foremost headache and disturbance of consciousness while seizures, brainstem/cerebellar signs and vomiting/nausea were rare compared to patients with solid malignancies.

In patients with a solid malignancy the most frequent affected cranial nerves were the vestibulochochlear nerve (7 patients) and optic nerve (7 patients) followed by facial nerve (4 patients), oculomotor nerve (4 patients), abducens nerve (4 patients), and vagus nerve (2 patients). Trigeminal nerve, hypoglossal nerve, and glossopharyngeal nerve affection were found in one patient each.

The facial nerve was the predominantly affected cranial nerve in patients with lymphoid malignancies (9 patients) followed by abducens nerve (6 patients), trigeminal nerve (4 patients), oculomotor nerve (3 patients), optic nerve (3 patients), vestibulocochlear nerve (2 patients), and hypoglossal nerve (one patient) impairment.

Symptoms and signs of spinal cord involvement were found in every fourth patient with a solid malignancy and hematological malignancy.

### CSF Findings

CSF analysis revealed an elevated CSF cell count in 60/76 patients (79%) with solid malignancies and in 34/37 patients (92%) with hematological malignancies (Table 3). All patients with myeloid malignancies had an elevated CSF cell count. The median CSF cell count for solid malignancies was 33 cells/μl (range: 1–831 cells/μl) and 80 cells/μl (range: 1–49,501 cells/μl) for hematological malignancies.

**Table 3:** CSF standard parameter findings of patients with leptomeningeal metastasis.

**Characteristics**	**Pleocytosis (≥5 cells/μl)**	**Elevated protein (≥500 mg/l)**	**Elevated Lactate (≥3.5 mmol/l)**	**Blood-CSF-barrier dysfunction**
Solid malignancies (*n* = 76)	79%	82%	68%	80%
Breast cancer (*n* = 26)	73%	88%	77%	91%
Lung cancer (*n* = 25)	80%	86%	55%	77%
Gastrointestinal cancer (*n* = 12)	75%	58%	70%	58%
Other malignancies (*n* = 13)	92%	83%	73%	77%
Hematological malignancies (*n* = 37)	92%	89%	48%	92%
Lymphoid malignancies (*n* = 31)	90%	87%	56%	90%
Myeloid malignancies (*n* = 6)	100%	100%	17%	100%
*p* value	0.03	0.57	0.08	0.16

Elevated CSF lactate levels (≥ 3.5 mmol/l) were detected in in 44/65 patients (68%) with solid malignancies (median CSF lactate level 4.5 mmol/l, range 1.7–13.3 mmol/l) and in 16/33 patients (48%) with hematological malignancies (median CSF lactate level: 2.8 mmol/l, range 1.1–9.7 mmol/l).

CSF glucose levels were available in 43/76 patients (57%) with solid malignancies (median CSF glucose level 2.2 mmol/l, range 0.5–6.5 mmol/l) and in 20/37 patients (54%) with hematological malignancies (median CSF glucose level 2.8 mmol/l, range 0.5–7.9 mmol/l).

CSF total protein was elevated in 58/71 patients (82%) with solid malignancies (median CSF total protein 1,094 mg/l, range 255–13,790 mg/l) and in 33/37 patients (89%) with hematological malignancies (median CSF total protein 928 mg/l, range 184–6,095 mg/l).

A blood-CSF barrier dysfunction as measured by QAlb was found in 55/69 patients (80%) with solid malignancies and in 33/36 patients (92%) with hematological malignancies. Barrier dysfunction was mild in 16/55 patients (29%) with solid malignancies and in 14/33 patients (42%) with hematological malignancies, moderate in 11/55 patients (16%) with solid malignancies and in 8/33 patients (21%) with hematological malignancies, and severe in 28/55 patients (55%) with solid malignancies and in 11/33 patients (33%) with hematological malignancies.

Oligoclonal bands restricted to CSF were found in 20/67 patients (30%) with solid malignancies and in 9/34 patients (26%) with hematological malignancies (Table 4).

**Table 4:** CSF immunology findings of patients with leptomeningeal metastasis.

**Characteristics**	**Intrathecal synthesis**			**CSF oligoclonal bands**	**CSF oligoclonal bands + MRZ reaction**
	**lgG**	**lgM**	**lgA**		
Solid malignancies (*n* = 76)	9%	11%	6%	30%	0%
Breast cancer (*n* = 26)	4%	5%	5%	27%	0%
Lung cancer (*n* = 25)	14%	14%	10%	38%	0%
Gastrointestinal cancer (*n* = 12)	17%	25%	8%	33%	0%
Other malignancies (*n* = 13)	0%	0%	0%	17%	0%
Hematological malignancies (*n* = 37)	0%	16%	11%	26%	0%
Lymphoid malignancies (*n* = 31)	0%	19%	10%	29%	0%
Myeloid malignancies (*n* = 6)	0%	0%	17%	17%	0%
*p* value	0.09	0.54	0.46	0.81	1.0

An intrathecal synthesis of IgM, IgG, or IgA according to Reiber-Felgenhauer graphs was found in 11/68 patients (16%) with solid malignancies and in 9/37 patients (24%) with hematological malignancies. An isolated IgG synthesis was detected in 4 and an isolated IgA synthesis in 3 patients with solid malignancies, while an isolated IgM synthesis did not occur. Two patients with solid malignancies had the combination of an intrathecal IgG, IgM, and IgA synthesis and another two patients the combination of an intrathecal IgM and IgA synthesis. In patients with hematological malignancies an intrathecal synthesis of IgG was not found. An isolated intrathecal synthesis of IgM was detected in 5 patients (of which no patient had a primary CNS lymphoma) and of IgA in 3 patients with hematological malignancies. The combination of an intrathecal synthesis of IgM and IgA occurred only in one patient with a hematological malignancy (primary CNS lymphoma).

MRZ reaction was investigated in 17/20 oligoclonal band positive patients (85%) with solid (of which 5 patients had an intrathecal IgG synthesis according to Reiber's graph) and in 7/9 oligoclonal band positive patients (77%) with hematological malignancies. None of these patients exhibited a positive MRZ reaction.

Of the 13 patients with solid malignancies and normal CSF cell count, 9 patients had elevated CSF total protein, 8 patients showed a blood-CSF barrier dysfunction, and 2 patients had elevated CSF lactate levels.

CSF parameters within the normal range (cell count, lactate, protein, QAlb, oligoclonal band status, intrathecal synthesis of immunoglobulins) were found in only 4 patients with solid malignancies, while all patients with hematological malignancies showed at least one of these parameters pathologically changed.

CSF flow cytometry as an additional diagnostic method to detect malignant cells was performed in 19 of the 37 patients with hematological malignancies (17 patients with lymphoid and 2 patients with myeloid malignancies) and confirmed a malignancy in all of these patients. Additionally, malignancies was confirmed by biopsy of lymphoid tissue in 4 patients and by immunochemistry in 3 patients. In one patient with acute myeloid leukemia NPM1 mutation in CSF tumor cells was found and in one patient with lymphoma additional molecular genetic testing showed monoclonal rearrangement in immunoglobulin heavy chain gene in CSF lymphocytes.

### MRI Findings and Correlation of MRI Findings and CSF Findings

MRI findings were extracted from patient records. MRI examinations were available in 109 patients: cranial MRI in 42/73 patients (58%) with solid malignancies and in 25/36 patients (69%) with hematological malignancies, spinal MRI in 1/73 patient (1%) with a solid malignancy and in 2/36 patients (6%) with hematological malignancies and both cranial and spinal MRI in 30/73 patients (41%) with solid malignancies and in 9/36 patients (25%) with hematological malignancies. In four patients MRI was not possible due to their pacemaker or disease severity. MRI protocols included T1-weighted, T2-weighted, and contrast-enhanced T1-weighted sequences. In the primary MRI reports signs of leptomeningeal metastasis in either cranial, spinal, or both examinations were described in 45/73 patients (62%) with solid malignancies and in 12/36 patients (33%) with hematological malignancies. Signs of cranial leptomeningeal metastasis were found in 25/72 patients (35%) with solid malignancies and in 7/34 patients (21%) with hematological malignancies, spinal leptomeningeal metastasis in 13/31 patients (42%) with solid malignancies and in 2/11 patients (18%) with hematological malignancies, and both cranial and spinal leptomeningeal metastasis in 7/30 patients (23%) with solid malignancies and in 3/9 patients (33%) with hematological malignancies. CNS parenchymal metastases were found in 40/73 patients (55%) with solid malignancies of which 22/40 patients (55%) presented leptomeningeal enhancement. In patients with hematological malignancies parenchymal infiltration of the CNS occurred in 16/36 patients (44%).

**Figure 2:**
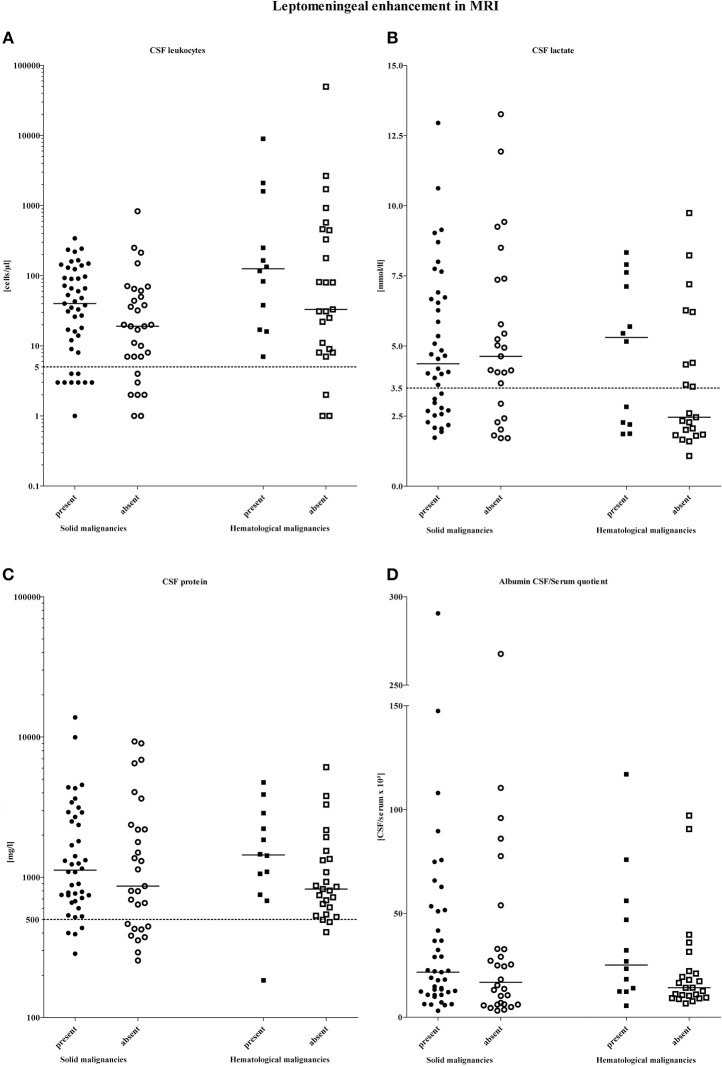
Distribution of CSF parameters according to the presence and absence of leptomeningeal gadolinium enhancement on MRI and stratified by the type of malignancy. Horizontal bars indicate the medians. The dashed lines on each graph show the upper limit of normal: **(A)** CSF cell count < 5 cells/μl, **(B)** CSF lactate levels < 3.5 mmol/l, **(C)** CSF protein levels < 500 mg/l, **(D)** the upper limit of normal of the CSF-serum albumin quotient is age-adjusted calculated).

**Figure 3:**
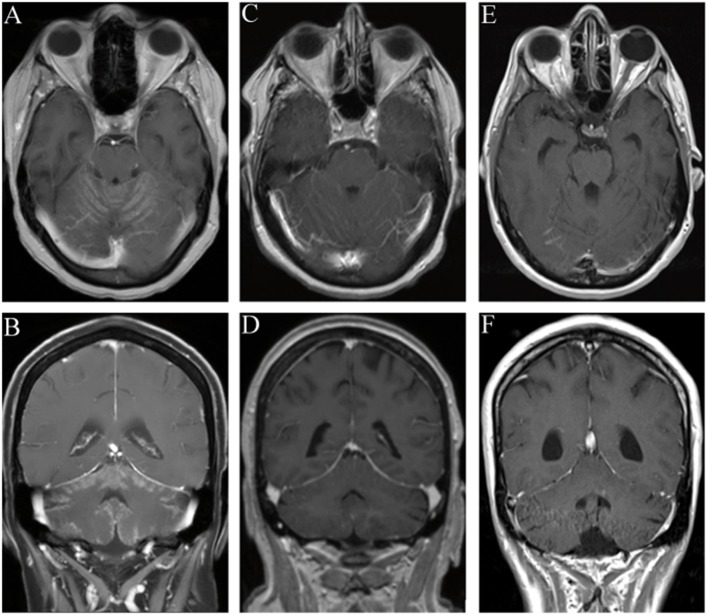
Representative MRI findings of patients with leptomeningeal metastasis. In all patients malignant cells were found in the CSF. **(A,B)** Gadolinium-enhanced coronal and axial T1-weighted images demonstrate leptomeningeal enhancement. CSF diagnostic revealed pleocytosis with 12 cells/μl. **(C,D)** Leptomeningeal enhancement in Gadolinium-enhanced coronal and axial T1-weighted images in a patient with normal CSF cell count. **(E,F)** No leptomeningeal enhancement could be detected in Gadolinium-enhanced coronal and axial T1-weighted images in a patient with CSF pleocytosis (7 cells/μl).

Although patients with hematological malignancies showed higher CSF cell counts, CSF lactate concentrations, CSF total protein levels, and QAlb values in cases when MRI demonstrated signs of leptomeningeal metastasis, differences were not significant. Patients with solid malignancies and leptomeningeal enhancement on MRI similarly showed a tendency of higher CSF cell counts, CSF total protein levels, and QAlb values (Figure 2). Figure 3 exemplifies representative MRI findings of patients of this study.

## Discussion

Leptomeningeal enhancement in MRI is likely caused by extravasation of contrast agent due to leakage in damaged vessels of the pia mater (25–28). Previous studies have also indicated that the presence of contrast enhancement of the leptomeninges is dependent on the origin of malignant cells in CSF (11). Due to their biological characteristics tumor cells of epithelial origin are more likely to adhere to the meninges and build layers of neoplastic tissue which can be detected by MRI (11, 29, 30). In line with other studies, we detected a higher rate of leptomeningeal contrast enhancement in MRI in patients with solid malignancies (61%) than in patients with hematological malignancies (33%). The lower sensitivity of MRI for detection of leptomeningeal metastasis caused by hematological malignancies is in line with previous studies and conclusively explained by the assumption that lymphoma or blast cells will rather float freely in CSF than adhere to meninges (11, 31–33).

A dominant adherence effect of epithelial tumor cells could also be an explanation for the lower CSF cell counts in patients with solid malignancies compared to hematological malignancies found in our study (11). Since 21% of patients with lymphoid malignancies and 8% patients with solid malignancies had normal cell counts, our observations support the recommendation of a thorough cytological examination of every CSF sample even when the CSF cell count is normal. The few available previous studies that included patients with cytologically proven leptomeningeal metastasis demonstrated similar proportions of patients with normal CSF cell count (34, 35).

Considering the different adherence effect of tumor cells to the leptomeninges, it seems conceivable that the presence of leptomeningeal enhancement could be related to CSF findings. In neurosarcoidosis for instance, CSF parameters have been demonstrated to correlate significantly with the presence of leptomeningeal enhancement in MRI (36). QAlb which indicates an increase of blood derived proteins in the CSF due to blood-CSF-barrier dysfunction has been described to be positively correlated to the presence of leptomeningeal enhancement in patients with aseptic meningitis (28). In our study, however, we did not find a significant correlation between the presence of leptomeningeal enhancement and blood-CSF-barrier dysfunction measured by QAlb. Likewise the standard CSF parameters cell count, lactate concentration and CSF protein level did not differ between patients with and without leptomeningeal enhancement shown by MRI.

Our data and prior studies indicate that CSF diagnostic should be performed regardless of MR imaging to obtain CSF parameters which could be useful as potential biomarkers. Herrlinger et al. demonstrated that the severity of the blood-CSF-barrier dysfunction measured by QAlb can serve as a prognostic marker for the disease course (7). The blood-CSF-barrier dysfunction in leptomeningeal metastasis is most likely caused by reduced CSF absorption due to obstruction by malignant cells (14, 34). Consequently hydrocephalus is a well-known and often fatal complication in patients with leptomeningeal metastasis which requires neurosurgical intervention (14). However, it would be interesting to investigate a correlation between QAlb and special flow sensitive MRI sequences in future studies.

CSF lactate has been demonstrated as another therapy-independent predictor of poor survival in patients with leptomeningeal metastasis (7). Moreover, elevated CSF lactate ≥ 3.5 mmol/l which was found in 48% of our patients with leptomeningeal metastasis due to lymphoid malignancies can also be useful for differential diagnosis when malignant cells are only suspected. Infectious disease including neuroborreliosis and viral meningoradiculitis can have a similar clinical presentation and CSF cytology like a lymphoid malignancy while elevated lactate levels are observed in <10% of these patients (34, 37).

Since the discrimination between leptomeningeal metastasis caused by lymphoid malignancies and primary inflammatory diseases can be challenging, humoral CSF parameters including intrathecal immunoglobulin synthesis or interleukin levels had been in focus as additional markers to identify lymphoma patients (34, 38). Although studies have demonstrated that these parameters are not suitable as additional diagnostic criteria for leptomeningeal metastasis, oligoclonal bands in CSF as evidence of an intrathecal immunoglobulin synthesis have been found in 30–40% of patients with leptomeningeal metastasis in previous studies (39–44). The origin of this intrathecal IgG is discussed controversially. One explanation might be that IgG is locally produced by perivascular plasma cells or activated B lymphocytes within meningeal tumor (40). In patients with leptomeningeal malignancies due to lymphoid malignancies some authors speculated that lymphoma cells might produce immunoglobulins or proteins mimicking immunoglobulins (42). However, we found a comparable prevalence of oligoclonal bands in patients with leptomeningeal metastasis regardless of the origin of malignant cells in CSF and in line with previous reports (41–45).

Another important observation of our study is that we did not observe any evidence of a polyspecific humoral immunoresponse in patients with leptomeningeal metastasis. Our results underline the significance of a positive MRZ reaction as a possible marker for multiple sclerosis rather than CNS autoimmunity in general (22, 46).

## Conclusion

CSF examination should be included in the diagnostic work-up for leptomeningeal metastasis especially when no signs of leptomeningeal metastasis can be found by MRI. CSF cytology is always mandatory regardless of CSF cell count and can be crucial even when leptomeningeal metastasis is not suspected.

## Data Availability

The datasets generated for this study are available on request to the corresponding author.

## Ethics Statement

The investigation was approved by the local Ethics Committee of the Hannover Medical School. Figures 3E,F were used with courtesy of Röntgenpraxis am Marstall. This is a retrospective study and only data were included that were evaluated for patients' treatment.

## Author Contributions

LB collected the data, participated in the design of the study, analyzed data, and drafted the manuscript. NM, JA, and WP analyzed data. UW, PR, K-WS, and MS analyzed data and contributed in drafting the manuscript. TS analyzed data and drafted the manuscript. PS conceived the study, analyzed data and drafted the manuscript.

### Conflict of Interest Statement

The authors declare that the research was conducted in the absence of any commercial or financial relationships that could be construed as a potential conflict of interest.

## Abbreviations

LM: Leptomeningeal metastasis
CNS: central nervous system
CSF: cerebrospinal fluid
MRI: magnetic resonance imaging
QAlb: CSF-serum albumin quotient
CUP: Cancer of unknown primary.

## Appendix

### Analytical Procedures

CSF and corresponding serum samples underwent standard diagnostic procedures in the neurochemistry laboratory of the Department of Neurology (15). Cells in the CSF were counted manually with a Fuchs-Rosenthal counting chamber. CSF cell count > 4 cells/μl were considered to be elevated. High volumes of CSF increase the chance of detecting malignant cells. For enrichment of cells 2–15 ml of CSF were precentrifuged at 145 g for 15 min. The cell sediment was resuspended in 0.2 ml cell culture medium and cytospins were prepared in a Shandon Cytospin 3 device at 90 g for 10 Min (16). Air dried cells were then stained with the Pappenheim method, a combination of May-Grünwald (Merck, Darmstadt, Germany) and Giemsa staining (Sigma-Aldrich, St.Louis, USA) (17). Cell differentiation was performed by microscopic examination of CSF samples by cytologists of the Department of Neurology. Standard criteria for malignancy such as abnormal size, form, and staining of cells and nucleus were applied (18). A Bradford dye-binding procedure was used to determine CSF total protein (cut-off = 500 mg/l). CSF lactate and CSF glucose were determined enzymatically (CSF lactate cut-off = 3.5 mmol/l). Albumin, IgG, IgM, and IgA in serum and CSF were measured nephelometrically by latex enhanced assay (Beckman Coulter IMMAGE).

Blood–CSF barrier function was evaluated by CSF-serum albumin quotients (QAlb). The age-adjusted upper reference limit of QAlb was calculated using the formula QAlb = 4 + (age in years/15) (19). A mild blood-CSF barrier dysfunction was defined as QAlb <15, a moderate as QAlb 15-25 and a severe as QAlb >25. Intrathecal synthesis of IgG, IgA, and IgM was calculated according to Reiber's revised hyperbolic function referring IgG, IgA, and IgM quotients to QAlb (19). Intrathecal synthesis of antibodies against measles virus, rubella virus, and varicella zoster virus, the so called “MRZ reaction,” was calculated according to the formula: (CSF virus antibody IgG/serum virus antibody IgG)/(CSF IgG total/serum IgG total) (20, 21). In case of an intrathecal IgG synthesis, the upper limit of the Reiber's hyperbolic function for IgG (Qlim IgG) instead of CSF IgG total (22) was employed. CSF-specific oligoclonal bands were determined by isoelectric focusing in polyacrylamide gels with consecutive silver staining (23). For all protein analyses, CSF and serum samples were analyzed within the same analytical series. At least 6 ml of CSF were obtained in all our patients by lumbar puncture. All CSF samples were processed and analyzed in our neurochemistry laboratory within 1 day after they were obtained. All methods are quality assured by participating in external quality control programs, the CSF survey of INSTAND (24).
